# On the Use of Generalized Coordinates to Describe the Temperature Dependence of Viscosity and Relaxation Time in the Glass Transition Region

**DOI:** 10.3390/ma19132814

**Published:** 2026-07-02

**Authors:** Alexey A. Mashanov, Irina V. Razumovskaya, Michael I. Ojovan, Yulia A. Batischeva, Migmar V. Darmaev

**Affiliations:** 1 Department of General and Theoretical Physics, Banzarov Buryat State University, Ulan-Ude 670000, Russia; mashanov@bsu.ru (A.A.M.); darmaev@bsu.ru (M.V.D.); 2 Department of Theoretical Physics, Moscow State Pedagogical University, Moscow 119435, Russia; irinarasum9@mail.ru (I.V.R.); yufi26@list.ru (Y.A.B.); 3 School of Chemical, Materials and Biological Engineering, The University of Sheffield, Sheffield S1 3JD, UK; 4 Department of Radiochemistry, Lomonosov Moscow State University, Moscow 119991, Russia; 5 Faculty of Fundamental Physical and Chemical Engineering, Lomonosov Moscow State University, Moscow 119991, Russia; 6 Institute of Physical Materials Science, Siberian Branch of the Russian Academy of Sciences, Ulan-Ude 670047, Russia

**Keywords:** viscosity, relaxation time, glass transition, Williams–Landel–Ferry (WLF) equation, Vogel–Fulcher–Tammann (VFT) equation, principle of corresponding states, glass transition activation energy, fragility

## Abstract

It is shown that the empirical Williams–Landel–Ferry (WLF) and Vogel–Fulcher–Tammann (VFT) equations, as well as the semiempirical equations of other researchers, for the viscosity η in the glass transition region are, in fact, hyperbolic functions of temperature with corresponding relationships between their parameters. The hyperbolic dependence can be derived from the mathematically common expansion of ln*η* into a Taylor series in a small temperature parameter near the glass transition temperature, provided that the temperature range satisfies approximately (*T* − *T*_g_)/*T*_g_ ≲ 0.1–0.15). The applicability of the principle of corresponding states for glasses of similar composition follows from this expansion. A new two-parameter equation in the form of a second-degree polynomial is proposed for ln(*η*) in the glass transition region. This equation contains physically significant parameters and adequately describes the available experimental data for individual glass-forming substances. The temperature range over which the proposed series expansion up to the third (quadratic in the small parameter) term is valid can be determined only experimentally, because the coefficients of the series depend on the nature of the glass. For the specific experimental data we used, the sharp temperature dependence of the viscosity in the glass transition region makes the quadratic polynomial applicable over almost the entire temperature range studied.

## 1. Introduction

Viscosity is a property of fundamental importance both for the molten and solid states of materials, quantifying the resistance of materials to flow and indicating the ability to dissipate momentum [1,2,3]. For example, in glass technology the viscosity determines melting, working and annealing temperatures, rate of refining, maximum use temperature, and crystallization rate, while in nature it governs the behavior of Earth’s mantle and volcanic lava flow rates [4,5,6,7,8,9,10]. The viscosity of glass-forming liquids and amorphous solids exhibits a characteristic and often dramatic temperature dependence that has motivated decades of theoretical and experimental investigation. Understanding the microscopic origins of viscous flow is essential not only for fundamental condensed-matter physics but also for the processing, stability, and long-term performance of technological glasses. Early physically motivated descriptions of viscosity focused on the structural rearrangements required for flow in network-forming materials. Mott proposed that viscous flow in vitreous silica is governed by the thermally activated rupture of Si–O bonds, with the number of broken bonds increasing with temperature and enabling local structural rearrangements [11]. This model established the principle that flow in network glasses is controlled by the energetics of bond rupture rather than by collective configurational entropy. A complementary structural interpretation was introduced earlier by Douglas, who proposed that the bridging oxygen atom between two silicon atoms can occupy two metastable positions separated by an energy barrier [12]. The role of defects in mediating viscous flow was further elaborated by Doremus, who demonstrated that amorphous silica contains SiO molecular defects and other structural irregularities that act as flow units [13]. These ideas were unified in the Douglas–Doremus–Ojovan (DDO) model, developed in detail in [14] and expanded to account for the effects of radiation on viscosity in [15,16], which has been successfully applied to various complex-nature multicomponent systems including industrial and nuclear-waste glasses, as demonstrated in [17,18,19,20,21]. Parallel to these structural models, thermodynamic theories have sought to relate viscosity to configurational entropy [22] or free volume [23]. Energy-landscape approaches, pioneered by Goldstein [24] and later developed by Stillinger and Weber [25], further emphasized the role of the multidimensional potential-energy surface in determining relaxation and flow. The topological constraint theory (TCT) treats the glass network as a mechanical truss whose rigidity is determined by the number and type of atomic constraints [26,27] so that viscosity and relaxation are governed by the balance between constraints and degrees of freedom. This perspective was followed by the description of the glass transition as a topological phase transition in disordered systems [28] based on the Kantor–Webman theorem, which established that “the rigidity threshold of an elastic percolating network is identical to the percolation threshold” and hence provides an explanation of ductile to brittle transition in vitrification, which is a feature of the calorimetric glass transition [29], later extended to real glass-forming systems, linking topological defect formation to viscosity and relaxation [30,31]. Most recently, the topological description has been refined by adding a generic criterion of melting based on the mathematical set theory, which assigns to bonding systems of solids a Hausdorff–Besicovitch dimensionality *D* = 3 and to bonding systems of melts a fractal one, *D* = 2.5 [32,33]. These studies reinforce the idea that viscosity is fundamentally controlled by the topological state of the network, with flow occurring through the creation, annihilation, and migration of topological defects. Zaccone et al. proved that defects are mediators of plasticity in amorphous solids [34].

Against this historical and theoretical background, the widespread use of empirical and semiempirical viscosity equations—most notably the Williams–Landel–Ferry (WLF) [35] and Vogel–Fulcher–Tammann (VFT) [36,37,38] equations—can be understood in a new light. Although often treated as purely empirical, these relations encode the same underlying physics: the temperature dependence of structural degrees of freedom, defect populations, and network rigidity. We show below that both WLF and VFT can be expressed as hyperbolic functions of temperature, emerging naturally from a universal Taylor expansion of ln(*η*) near the glass transition temperature. This expansion clarifies the relationships among the parameters of WLF, VFT, and related equations, and it provides a theoretical basis for the principle of corresponding states in glass viscosity. Building on this insight, we introduce a new two-parameter quadratic expression for ln(*η*) in the glass transition region. This equation contains physically meaningful parameters, is consistent with the structural, thermodynamic, and topological models discussed above, and accurately describes available experimental data for a wide range of glass-forming substances. The goal of this paper is hence to demonstrate that the known empirical relationships can be treated, within a limited temperature window near *T_g_*, as special cases of a more general approach based on the Taylor series expansion of the logarithm of viscosity near the glass transition temperature, and to propose a new two-parameter equation whose coefficients have a direct physical meaning. We explicitly restrict our analysis to the range (*T* − *T_g_*)/*T_g_* ≲ 0.15, where the quadratic truncation of the series gives the same result as the WLF equation.

## 2. Modelling the Temperature Dependence in the Glass Transition Region

To describe the temperature dependence of viscosity *η* or relaxation time τ in the glass transition region, two empirical equations are most often used: the Williams–Landel–Ferry (WLF) equation:

(1) $$\ln a_{T}=-C_{1}\frac{T\;-\;T_{g}}{T\;-\;T_{g}\;+\;C_{2}};\;a_{T}=\frac{\tau \left(T\right)}{\tau \left(T_{g}\right)}\;≅\;\frac{\eta \left(T\right)}{\eta \left(T_{g}\right)},$$

where *C*_1_ and *C*_2_ are empirical constants, *T_g_* is the glass transition temperature, and *τ* is the relaxation time at temperature *T* [35]; and the Vogel–Fulcher–Tammann (VFT) equation:

(2) $$\ln a_{T}\;=\;\left(\frac{B_{0}}{T\;-\;T_{0}}\;-\;\frac{B_{0}}{T_{g}\;-{\;T}_{0}}\right),$$

where *B*_0_ and *T*_0_ are empirical constants [36,37,38].

Recently, a number of empirical equations have been proposed ([39,40,41], etc.). In particular, for metallic glasses, a hyperbolic dependence of ln*a_T_* on temperature and the fulfillment of the principle of corresponding states have been demonstrated if the value of *T_A_*/*T* is plotted on the abscissa axis as the temperature parameter, where the temperature *T_A_* is some significant physical parameter [41]. The equation proposed in [41] for viscosity (in decimal logarithms) has the following form:

(3) $$\log\eta \;=\;\log\eta_{0}\;+\;T_{A}/T.$$

For ln*a_T_* this gives

(4) $$\ln a_{T}=\;\alpha \left(\frac{T_{A}}{T}-\frac{T_{A}}{T_{g}}\right),$$

where *α* ≈ 2.3026 is the conversion factor from decimal to natural logarithms.

The purpose of this work is to show that the known empirical dependencies can be derived from a general mathematical approach based on the decomposition of the logarithm of viscosity into a Taylor series near the glass transition temperature. Thus, the common mathematical origin of the empirical WLF and VFT forms, independent of the specific physical mechanism, is mathematically substantiated. At the same time, it becomes possible to use an alternative two-parameter equation to describe experimental data, the coefficients of which have a direct physical meaning.

**Figure 1:**
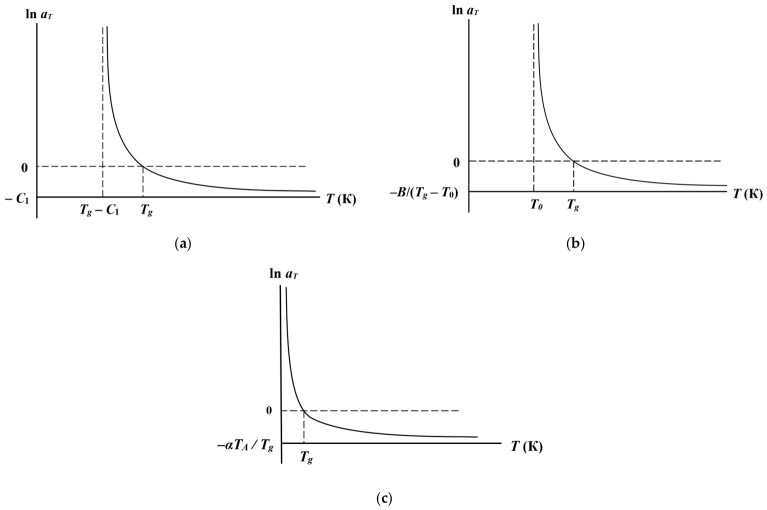
Graphs of the ln*a_T_*(*T*) function: (**a**)—according to the WLF equation, which can be represented as $\ln a_{T}=-C_{1}\frac{T\;-{\;T}_{g}}{T\;-\;T_{g}\;+\;C_{2}}=-C_{1}\left(1\;-\;\frac{C_{2}}{T\;-\;T_{g}\;+\;C_{2}}\right)$; (**b**)—according to the VFT Equation (2); (**c**)—according to Equation (4) in accordance with Equation (3).

For a given glass transition temperature *T_g_*, the experimental curve ln*a_T_*(*T*) can be described by both the WLF equation and the VFT equation. Each equation contains, in addition to *T_g_*, two “fitting” parameters. At the same time, simple algebraic relations between the empirical constants of these equations can be established from their graphical interpretation of the WLF and VFT equations (Figure 1). It becomes clear that both empirical equations correspond to the hyperbolic dependence on temperature; they give hyperbolas shifted with respect to the abscissa and ordinate axes. The magnitude of these shifts makes it possible to visually compare the constants of the WLF and VFT equations: *C_1_* = *B_0_*/(*T_g_* − *T_0_*); *T_g_* − *C_2_* = *T_0_*. Comparison of the authors’ equation in [41] and WLF leads to the equality of the coefficient *C_1_* = *αT_A_*/*T_g_* with *C_2_* = *T*_g_. Comparison of the VFT equation and (4) gives *T_0_* = 0 and *B_0_* = *αT_A_.*

We do not attempt here to discuss the specifics of vitrification for various glass-forming systems. For metallic glasses, only Equation (3) and the corresponding graph in Figure 1c are presented as illustrations.

## 3. Theoretical Background of Empirical Equations: Taylor Series Expansion

It is well known that the Taylor series expansion of a function in a small parameter is widely used in physics. For example, in solid state physics, the universality of Hooke’s law is proved from the mathematical universality of the Taylor series as applied to the decomposition of the energy of an atom (molecule) in a solid body by the deformation of an interatomic bond as a small parameter.

In the case of ln*η*, the series expansion can be carried out near *T_g_* in terms of a small parameter *δ* = (*T* − *T_g_*)/*T_g_*:

(5) $$\ln\eta (T)=\ln\eta \left(T_{g}\right)\;+\;A\delta +B\delta^{2}\;+\cdots$$

where $A\;=\;{\left.\frac{dln\eta }{d\delta }\right|}_{T_{g}}\;=\;{T_{g}\left.\frac{dln\eta }{dT}\right|}_{T_{g}}$, $B\;=\;\frac{1}{2}{\left.\frac{d^{2}ln\eta }{{d\delta }^{2}}\right|}_{T_{g}}\;=\;\frac{1}{2}{T_{g}^{2}\left.\frac{d^{2}ln\eta }{{dT}^{2}}\right|}_{T_{g}}$; at the same time, *A* < 0 (as temperature decreases, viscosity increases).

Restricting ourselves to the quadratic term in *δ* in the expansion, we obtain

(6) $$\ln a_{T}\;=\ln\frac{\eta \left(T\right)}{\eta \left(T_{g}\right)}\approx A\delta +B\delta^{2},$$

The function ln*a_T_*(*δ*) can be well approximated by a second-degree polynomial (6) when the cubic and higher-order terms in the Taylor expansion are negligible. This convergence is favored by the steep variation of viscosity with temperature in the glass transition region.

**Figure 2:**
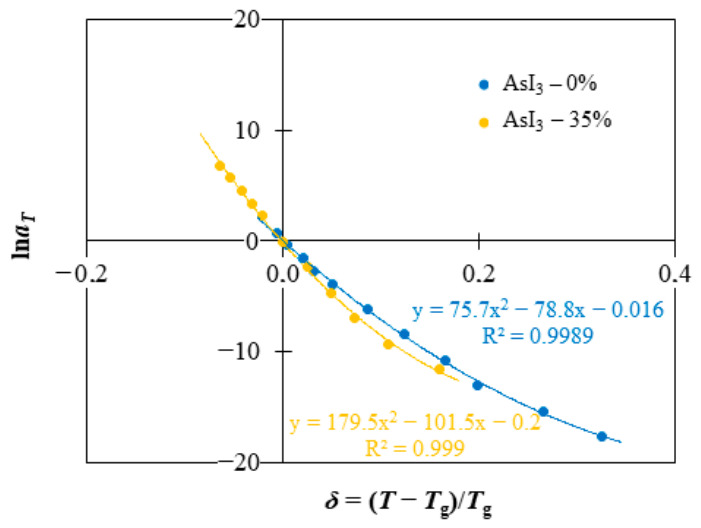
Experimental data for chalcogenide glasses As_2_S_3_–AsI_3_ with 0 mol% and 35 mol% AsI_3_ (symbols) and the corresponding second-degree polynomial fits (curves). Fitted coefficients: for 0% AsI_3_, *A* = −78.8, *B* = 75.7; for 35% AsI_3_, *A* = −101.5, *B* = 179.5.

**Figure 3:**
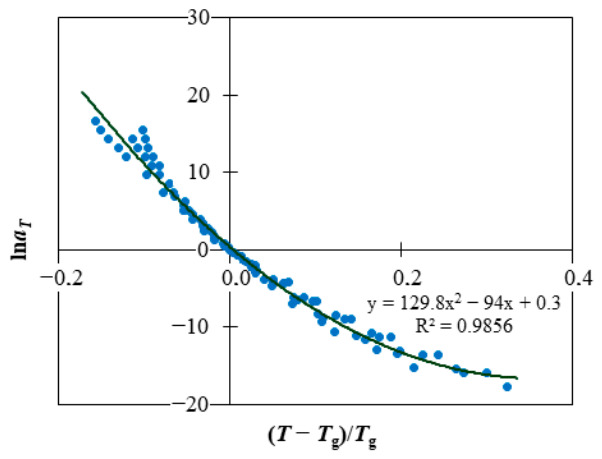
A second-degree polynomial (solid curve) describing all the experimental data for As_2_S_3_–AsI_3_ compositions in Table 1 in generalized coordinates (*T* − *T_g_*)/*T_g_* with coefficients *A* = −94; *B* = 129.8.

Figure 2 and Figure 3 show graphs of dependencies ln*a_T_*(*δ*) based on experimental data on glasses of different compositions and the corresponding polynomials of the second degree, which successfully describe these dependencies. Coefficients *A* and *B* are determined from the equations of polynomials of the second degree in MS Excel. Table 1 shows the values of coefficients *A* and *B* for a number of chalcogenide and silicate glasses. In this table, the coefficients *A* and *B* were determined by the above method, calculated according to graphs in coordinates (*T* − *T_g_*)/ln*a_T_*(*δ*) and (*T* − *T_g_*) from experimental data on the temperature dependence of viscosity [42].

The coefficients *A* and *B* in Equation (6) have a clear physical meaning: they are related to the first and second derivatives of the logarithm of viscosity by temperature at the glass transition point, and, consequently, to the effective activation energy and its temperature dependence [48].

Let us show that coefficient *A* is directly related to such an important characteristic of glasses as the fragility m according to Angell [49]:

(7) $$m={\left.\frac{\partial \mathrm{lg}\eta }{\partial \left(T_{g}/T\right)}\right|}_{T_{g}}.$$

Coefficient *A* according to (5) is equal to

(8) $$A={\left.\frac{d\ln\eta }{\mathrm{d\delta}}\right|}_{T_{g}}=\alpha {\left.\frac{d\mathrm{lg}\eta }{d(T_{g}/T)}\right|}_{T_{g}}\frac{d(T_{g}/T)}{d\delta }=-\alpha m;\;\;B=\frac{1}{2}{\left.\frac{d^{2}ln\eta }{{d\delta }^{2}}\right|}_{T_{g}}=\frac{1}{2}\frac{dA}{d\delta }=\frac{1}{2}\alpha T_{g}{\left.\frac{dm}{dT}\right|}_{T_{g}},$$

where *α* ≈ 2.3 is the coefficient of transition from decimal logarithms to natural logarithms.

Thus, the fragility is equal to

(9) $$m=-\frac{A}{\alpha },$$

In [50] it was shown that with sufficiently small values of the parameter *δ*

(10) $$\left|B\delta /A\right|\;\ll \;1,$$

one can use a certain approximation by putting

(11) $$\left(1+B\delta /A\right)\;\approx \;{\left(1-B\delta /A\right)}^{-1},$$

and convert Equation (6) into an equation analogous to the WLF equation:

(12) $$\ln a_{T}\;\approx \;A\delta \;+\;B\delta^{2}\;=\;A\delta \left(1\;+\;B\delta /A\right)\;\approx {\;A\delta \left(1\;-\;B\delta /A\right)}^{-1}\;=\;-\frac{A^{2}}{B}\frac{T\;-\;T_{g}}{T\;-\;T_{g}\;-\;AT_{g}/B}.$$

The resulting Equation (9) is identical to the experimental WLF equation, in which the coefficients are equal to

(13) $$C_{1}=\frac{A^{2}}{B},\;C_{2}=-\frac{AT_{g}}{B}$$

The relationship of the coefficients *A* and *B* with the coefficients of the VFT equation is easy to obtain from the above-mentioned relationship of the latter with the coefficients *C*_1_ and *C*_2_ [50,51].

Let us estimate the value of *δ*, i.e., the value of the relative temperature range (*T − T_g_*)/*T_g_*, in which the approximation (11) is valid, and from the Taylor series we can obtain the WLF equation.

At *Bδ*/*A =* 0.1; (1 + *Bδ*/*A*) = 1.1 and (1 *− Bδ*/*A*)^−1^ = 1.111.... With lower values of *Bδ*/*A*, the ratio (8) is even more fulfilled.

At *Bδ*/*A* = 0.2; (1 *+ Bδ/A)* = 1.2 and (1 *− Bδ*/*A*)^−1^ = 1.25 (the identification accuracy of the equations is about 4%).

The experimental values of the coefficients *A* and B in Table 1 make it possible to estimate the temperature range Δ*T* above and below *T_g_*, in which condition (11) is satisfied, and the second-degree polynomial (6) practically coincides with the hyperbolic dependence of WLF (and VFT). At the same time, we assumed that the temperature range ∆*T* includes temperatures both above and below the glass transition temperature |*Bδ*/*A*| = 0.1:

(14) $$∆T\;=\;0.2T_{g}\frac{\left|A\right|}{B}$$

The ∆*T* interval practically includes the entire temperature range of the experiments in Figure 2 and Figure 3.

Comparison of Equations (9) and (13) makes it possible to express fragility in terms of the coefficients *C*_1_ and *C*_2_ of the WLF equation:

(15) $$m\;=\;-\frac{C_{1}T_{g}}{\alpha C_{2}}$$

**Figure 4:**
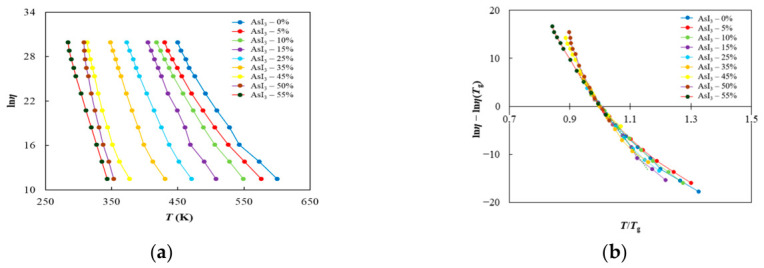
The temperature dependence of viscosity in the glass transition region: (**a**)—for chalcogenide glasses with different additive contents; (**b**)—the same data in generalized *T*/*T_g_* coordinates.

**Figure 5:**
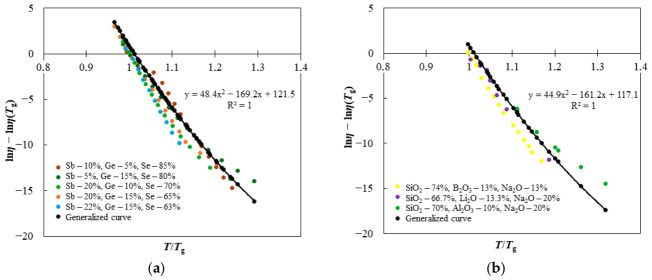
The temperature dependence of viscosity in generalized coordinates: (**a**)—for chalcogenide glasses of different compositions; (**b**)—for silicate glasses of three compositions. Solid curves are the result of averaging.

Experimental viscosity data in the field of glass transition for glasses of the same class can be quite successfully represented by a single curve in generalized *T*/*T_g_* coordinates (Figure 4 and Figure 5).

In Figure 4b and Figure 5a,b, it is enough to shift the coordinates on the abscissa axis by 1 to the left, i.e., move the point of intersection of the curve with the abscissa axis to the origin, and we get a dependence of ln*a_T_* not on *T*/*T_g_*, but on (*T*/*T_g_* − 1), i.e., on *δ*.

However, the curves do not coincide completely; the principle of corresponding states is not exact. Its strict validity would require identical coefficients A and B in Equation (6), because the argument in this equation is the reduced temperature *δ* = (*T − T_g_*)/*T_g_*. In fact, as can be seen from the data in Table 1, this is true only with small variations in the composition. Changes in composition change the structure of the glass-forming substance and its relaxation properties. However, the average values of *A* and *B* for this class of glasses may be of interest, as well as an average curve such as the one given in Figure 5.

Note that the criterion of the implementation of the principle of corresponding states, in accordance with Formula (8), must be a constant value of *mT_g_* (first of all) and the value of $T_{g}^{2}{\left.\frac{dm}{dT}\right|}_{T_{g}}.$

If for these glasses *A* and *B* change little, then in the WLF equation the coefficient *C*_1_ = *A*_2_/*B* is almost a constant value. Indeed, the coefficient *C*_1_ ≈ 40–60 (Table 1) and is weakly dependent on the nature of the glass [52].

The coefficient *C*_2_ = *−AT_g_*/*B*. With slightly changing values of *A* and *B*, *T* is proportional to the glasses of this series, *C*_2_, and the WLF Equation (1) corresponds to the law of corresponding states.

## 4. Conclusions

Applying the Taylor series decomposition to ln*η*(*T*) near the glass transition temperature using the relative temperature change *δ* = (*T − T_g_*)/*T_g_* as a small parameter yields a number of useful results, described below.

The obtained second-degree polynomial describes well the experimental viscosity data in the field of glass transition for a number of glass-forming systems. Polynomial coefficients, in contrast to the constants of empirical equations, have a physical meaning and are directly related to the effective activation energy of the vitrification process and to fragility.

It is shown that the well-known empirical equations WLF and VFT can be derived from this Taylor series for a temperature range near the glass transition temperature, and the universality of these equations is determined by the mathematical universality of this decomposition. This temperature range corresponds to *δ* ≲ 0.15. The relationship between the coefficients of the first terms of the series (polynomial coefficients) and the coefficients of the WLF and VFT equations has been established.

It is shown that the principle of corresponding states is realized for glasses similar in composition; the more accurate it is, the closer the values of the coefficients of the polynomial describing them. This condition can also be expressed as a requirement for the closeness of the fragility *m* and the product of its temperature derivative ${\left.\frac{dm}{dT}\right|}_{T_{g}}$ and *T_g_*.

## Figures and Tables

**Table 1:** Parameters of the VLF equation and coefficients *A* and *B* for some chalcogenide and silicate glassy systems based on experimental data [43,44,45,46,47].

Glass			*T_g_,* *K*	−*A*	*B*	Δ*T*, *K*	*C*_1_	*C*_2_ *,* *K*
mol%								
As_2_S_3_		AsI_3_	[43]					
100		0	453	78.8	75.7	94	54.7	305.8
95		5	443	74.0	70.5	93	50.2	285.8
90		10	431	76.6	67.1	98	53.0	274.4
85		15	418	97.0	117.9	68	58.8	248.7
75		25	394	90.0	107.4	66	55.1	229.6
65		35	372	101.5	179.5	42	58.6	234.6
55		45	353	86.9	326.3	19	-	-
50		50	343	96.7	486.9	14	-	-
45		55	337	91.5	78.4	78	-	-
Averaging:			-	94.0	129.8	-	-	-
Sb	Ge	Se	[44]					
10	5	85	351	50.1	63.0	56	23.9	204.4
5	15	80	408	72.1	82.8	71	36.9	191.6
20	10	70	424	93.8	150.4	53	43.2	200.3
20	15	65	489	85.9	125.6	67	47.5	271.9
22	15	63	520	94.4	80.7	122	36.8	270.8
Averaging:			-	81.8	108.2		-	-
SiO_2_	B_2_O_3_	Na_2_O	[45]					
74	13	13	868	91.3	120.7	131	44.0	395.4
SiO_2_	Li_2_O	Na_2_O	[46]					
66.7	13.3	20	656	68.7	24.7	365	63.1	431.2
SiO_2_	Al_2_O_3_	Na_2_O	[47]					
70	10	20	810	68.0	66.4	166	32.0	398.2
Averaging:			-	84.1	127.7		-	

## Data Availability

The original contributions presented in this study are included in the article. Further inquiries can be directed to the corresponding author.
